# Foraging flexibility and search patterns are unlinked during breeding in a free-ranging seabird

**DOI:** 10.1007/s00227-016-2826-x

**Published:** 2016-03-14

**Authors:** Akiko Shoji, Stéphane Aris-Brosou, Ellie Owen, Mark Bolton, Dave Boyle, Annette Fayet, Ben Dean, Holly Kirk, Robin Freeman, Chris Perrins, Tim Guilford

**Affiliations:** Department of Zoology, University of Oxford, Oxford, Oxfordshire UK; Department of Mathematics and Statistics, University of Ottawa, Ottawa, ON K1N 6N5 Canada; The Royal Society for the Protection of Birds, The Lodge, Sandy, Bedfordshire SG19 2DL UK; Edward Grey Institute of Field Ornithology, University of Oxford, Oxford, Oxfordshire UK; Institute of Zoology, Zoological Society of London, Regents Park, London, NW1 4RY UK

## Abstract

In order to maximize foraging efficiency in a varying environment, predators are expected to optimize their search strategy. Environmental conditions are one important factor affecting these movement patterns, but variations in breeding constraints (self-feeding vs. feeding young and self-feeding) during different breeding stages (incubation vs. chick-rearing) are often overlooked, so that the mechanisms responsible for such behavioral shifts are still unknown. Here, to test how search patterns are affected at different breeding stages and to explore the proximate causes of these variations, we deployed data loggers to record both position (global positioning system) and dive activity (time–depth recorders) of a colonial breeding seabird, the razorbill *Alca torda*. Over a period of 3 years, our recordings of 56 foraging trips from 18 breeders show that while there is no evidence for individual route fidelity, razorbills exhibit higher foraging flexibility during incubation than during chick rearing, when foraging becomes more focused on an area of high primary productivity. We further show that this behavioral shift is not due to a shift in search patterns, as reorientations during foraging are independent of breeding stage. Our results suggest that foraging flexibility and search patterns are unlinked, perhaps because birds can read cues from their environment, including conspecifics, to optimize their foraging efficiency.

**Electronic supplementary material:**

The online version of this article (doi:10.1007/s00227-016-2826-x) contains supplementary material, which is available to authorized users.

## Introduction

How animals adjust their foraging behavior in response to environmental changes is a fundamental question in the context of optimal foraging theory (Stephens and Krebs 1986). In particular, under the assumption that animals maximize net energy gain, and where animals forage in a patchy environment, the marginal value theorem (MVT) describes quantitatively how long an animal should keep searching before giving up and moving on to the next patch or returning to the nest (Charnov 1976; Orians and Pearson 1979). However, MVT has been developed and tested only in limited model systems characterized by small scale and low travel costs (Stephens and Krebs 1986). In actuality, foragers such as seabirds cover a large foraging range where prey is often scarce and unpredictably distributed (Lack 1968; but see Weimerskirch 2007). Furthermore, many central-place foragers not only commute between the colony and prey patches, but also load food for their young. Classical foraging theory predicts that prey load size should increase with the travel distance, but carrying prey to the young for longer distances increases the energetic expenditure of travel, which is therefore costly (Cuthill and Kacelnik 1990). The cost of transporting prey also depends on the loading modes, as the transport cost of carrying prey in the beak is energetically higher than carrying it internally. Thus, foraging behavior is determined by balancing trade-offs between energy expenditures and gains. Although central-place foraging models provide useful predictions concerning when animals should increase foraging distances, or how prey patch quality should influence this decision (Schoener 1979), it is still unclear how animals vary and optimize their search patterns in the wild.

The adoption of an efficient aerial search strategy is critical for successful foraging, especially as animals need to invest both in their own survival and in reproduction. Constraints are likely to be stage dependent because parents must commute frequently to feed young during rearing, while during incubation the pressure is low from a foraging perspective because birds only need to feed themselves (Jakubas et al. 2014). The constraints between the two stages, incubation and chick rearing, have been studied using bird-borne loggers. These studies showed that auks spend roughly twice as much time diving during the chick-rearing period, compared to the incubation period, and hence expend substantially more energy (Benvenuti et al. 2002; Ito et al. 2010; Elliott et al. 2014). This is particularly the case for razorbills, members of the auk family *Alcidae*, which are colonially breeding diving predators that feed mainly on fish during summer (Wanless et al. 1990). Compared to many other seabirds, auks have high flight costs because of their relatively high wing loading (Pennycuick 1987; Elliott et al. 2013). As a result, energy expenditure is expected to increase substantially with foraging distance, as has been shown in the closely related common guillemot *Uria aalge* (Gaston 1985). Because of the high energetic costs associated with commuting during breeding, razorbills may be under strong pressure to minimize transit costs (Thaxter et al. 2010; Elliott et al. 2013). Thus, we expect that incubating razorbills may expand their foraging area, flying further away from the colony and spending more time out at sea to find high-quality, but rare prey items, while chick-rearing birds may select lower-quality, but more predictably located prey items (Elliott et al. 2009).

To test these predictions, we here used an empirical approach based on data from three field seasons to assess how razorbills optimize their search strategy at two different breeding stages in an environment where resource availability changes during the season. More specifically, we hypothesized that razorbills will change aerial search strategy depending on their breeding stage, environmental conditions or both. To test this hypothesis, we compared changes in horizontal movement patterns by focusing on three descriptors of foraging: trip route similarity (memory), turning angle (change of bearing) and step length distribution (foraging strategy). By combining data from horizontal (global positioning system; GPS) and vertical (time–depth–temperature recorders; TDR) search patterns, we show that razorbills adopt search patterns that are surprisingly consistent over different breeding stages while adjusting flexibly their foraging locations in a changing environment.

## Methods

### Study site and field methods

Our observations were made at a razorbill colony on Skomer Island (54°44′N, 5°17′W), Wales, UK, during the three breeding seasons between 2011 and 2013. We successfully obtained data during the chick-rearing period in 2011 (*N* = 8 birds), incubation period in 2012 (*N* = 4 birds), and both incubation (*N* = 6 birds) and chick-rearing (*N* = 6 birds) periods in 2013. In all years, an adult from each nest was captured at the nesting crevice with a leg hook and fitted with two data logging devices: a GPS logger (unpackaged i-gotU GT-120: Mobile Action; mass of device = 12 g (<15 g including heat shrink; length = 43 mm; width = 24 mm; height = 9 mm; sampling interval = 5 min) and a time–depth–temperature recorder (TDR: Cefas Technology Ltd, Lowestoft, UK). We attached TDR loggers with either TESA marine cloth tape attached to the central four tail feathers (2011) or duct tape to a darvic leg ring cylindrically (2012, 2013; mass = 2.7 g; diameter = 1 cm; length = 3.3 cm; sampling interval for pressure = 1 s, temperature = 15 s). We also simultaneously secured GPS loggers with thin strips of tape that were attached to the back underlying a small number of contour feathers. Heat shrink plastic (model number = CLR-20/50, Finish Adapt Ltd, Swindon) was used to seal GPS loggers. The total mass of devices with attachment materials was less than 19 g (always <4 % of adult body mass), and feather attachments were designed to fall off naturally within 3 weeks if not retrieved. Handling time was usually <10 min and always <15 min. At the time of capture and recapture at the nest, we directly confirmed breeding status. Because of the small size of the accessible colony at this site and to minimize total disturbance, it was not possible to monitor nests intensively throughout the season or to compare potential impacts directly with controls. However, observationally birds appeared to behave normally at the colony and to complete foraging trips of typical duration (Hipfner and Chapdelaine 2002). Similar techniques employed on another (but smaller) seabird, the Manx shearwater *Puffinus puffinus*, did not lead to measurable impacts relative to controls on reproductive success, chick growth rates or foraging trip lengths (Dean et al. 2013).

### Data processing

GPS and TDR data were used to study horizontal and vertical movement patterns, respectively. Combining GPS tracks with TDR data, we derived time spent in flight, diving and on the water. Data processing and analysis were conducted in R version 2.15.2 (R Development Core Team 2014). All positional fixes were mapped using the Universal Transverse Mercator coordinate system. GPS-logged horizontal ground speed and TDR-logged temperatures were interpolated using cubic splines at a frequency of 1 Hz. Analysis of TDR-based dive data was conducted using the R package diveMove (Luque and Fried 2011), which corrected for device drift and extracted dives based on the bout-ending criterion (Mori et al. 2001). Only dives deeper than 1 m were considered, as shallower dives often occur during bathing or other activities that are not associated directly with foraging. Combined with pressure data from TDRs, lower speeds were considered to relate to drifting or foraging on the surface while higher values were interpreted as flight (Guilford et al. 2008). An appropriate speed threshold was selected based on the distribution of speeds (Fig. S1). Foraging trips were defined as trips made beyond a 1-km-radius area surrounding the colony. Primary productivity (chlorophyll *a*) data for the 2011–2013 breeding seasons were supplied by the NERC Earth Observation Data Acquisition and Analysis Service (www.neodaas.ac.uk). This environmental covariate is assumed to be a proxy for prey abundance and has previously been shown to be an important predictor of foraging behavior (Votier et al. 2010). Primary productivity data consisted of satellite images at a 500 × 500 m^2^ resolution taken three to five times a day and formatted to the Unidata’s netCDF. An R script supplied by NEODAAS was used to read these files and was modified to extract productivity data for each dive given its date and coordinates. Bathymetry was obtained from the US National Oceanic and Atmospheric Administration with the R package marmap (Pante and Simon-Bouhet 2013).

### Data analyses

Foraging behavior was analyzed in terms of inter- and intra-individual similarities and in terms of search strategies. First, route similarity, which gauges the potential use of memory and learning both within and between individuals (Guilford and Biro 2014), was analyzed by comparing foraging trips based on a nearest neighbor analysis (NNA; Freeman et al. 2011), which quantifies the spatial similarity among trips (see Supplementary Material for details). As we compare a focal trip to each of all other trips for several days (up to 7 days), if there are consistent patterns either within a day or between days, we should detect any pattern. To remove the effect of drift on the logging of horizontal movement, we have excluded resting periods from this analysis.

Linear mixed models (LMM) were employed to assess the impact of breeding stage on trip duration (hypothesis *H*_1_) and travel distances (*H*_2_); as data for multiple trips were collected for each individual bird, we accounted for pseudo-replication by including individual identity as a random effect in the models. Each hypothesis (*H*_1_ and *H*_2_) was assessed by fitting a model with a variable of interest (here, breeding stage) and with or without intercept only, our null model. Model choice was based on ΔAIC values, with |ΔAIC| > 2 standing as evidence against the null model (Burnham and Anderson 2004). Model fitting used the lmer() function in the package lme4 (Bates and Maechler 2009) in R. Foraging trip duration has been shown to differ between incubating and chick-rearing auks (Gaston and Jones 1998), as adults need to increase the frequency of visits to their nest when rearing chicks. As a result, we predicted that chick-rearing birds would focus their foraging on a particular area where productivity is high (a “region of interest” or ROI), as is the case in Manx shearwaters, another seabird breeding on Skomer Island (Shoji et al. 2015). To examine this question, we further investigated whether adults target such a ROI. To understand the reasons why birds would preferentially forage in a ROI, we tested whether there is an association between prey availability and foraging location both in space and in time. During the study period, razorbills exclusively brought sand eels (*Ammodytes*) to their young (Boyle, per. obs.). However, no data exist to quantify prey availability exactly when and where the birds foraged. As a proxy for resource availability (Eliasen et al. 2011), we extracted data on primary productivity (PP) for each bird at each dive location (12,761 dives in total). As a proxy for PP, we obtained chlorophyll Chl OC5 concentrations from NEODAAS (see above) at dive times and locations across the four conditions (outside vs. inside ROI, by breeding stage) and compared the corresponding densities (see Supplementary Materials for details).

While birds change their foraging behavior when a shift in PP is observed (see Results), we still do not know by which mechanism this adaptive behavioral response takes place. To test the hypothesis that this behavioral change reflects a change in aerial search pattern, from a random exploration of their environment (exponential patterns) to a power-law distribution (heavy-tail patterns), reorientation patterns during flight were analyzed separately for each breeding stage. As our GPS sampling rate was 5 min, all steps with a time interval >8 min were ignored (because these are likely to be due to GPS errors). Bearings *θ*, i.e., the angle between the colony and where birds either dived or flew, were calculated to gauge the general direction taken by birds for each kind of activity during each breeding stage (Fig. S2). Reorientations were then used to define steps and their associated length using Turchin’s method based on the change of angle *α* between three consecutive GPS logs (before interpolation; (Turchin 1998; de Jager et al. 2011, 2014). Two thresholds were examined to test the impact of mild (*α* = *π*/4, or 45°) and radical (*α* = 7*π*/8, or 157.5°) reorientations.

Exponential and power-law models were fitted to the complementary cumulative distribution of normalized step lengths by least square using the general model outlined by Edwards et al. (2007). Under this standard approach, the probability distribution of step lengths *l* is given by $(1 - \mu )l_{\mathrm{min} }^{1 - \mu }  l^{ - \mu }$, where $l_{\mathrm{min} }^{{}}$ is the minimum step length and *μ* is the scaling exponent. Minimum step lengths were set empirically to the minimum observed length as in de Jager et al. (2014). Scaling exponents between 1 and 3 correspond to heavy-tail movement while *μ* > 3 is the signature of exponential motion (de Jager et al. 2014). Only reorientations were considered.

## Results

### Foraging behavior clusters by breeding stage

GPS recorders logged a total of 56 foraging trips from 18 breeding razorbills (average of 3.1 ± 1.8 trips/individual, range 1–7). Visual inspection of individual routes suggested the existence of two foraging patterns: one with a dispersed foraging range, during incubation, and one with narrow and repeated routes, during chick rearing (Fig. S4).

**Fig. 1:**
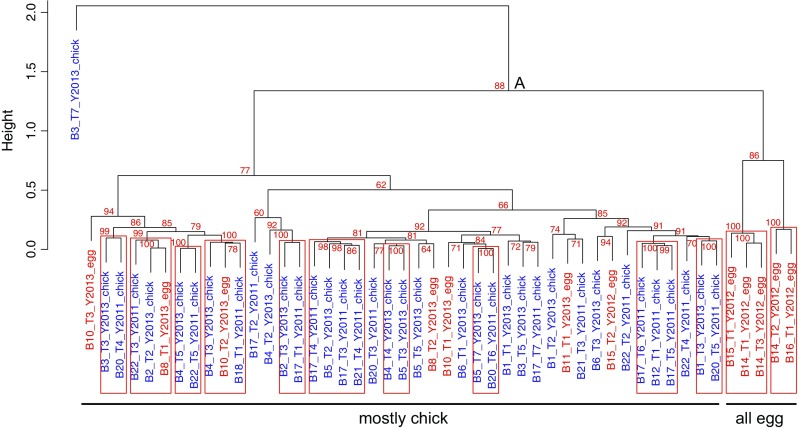
Hierarchical clustering of nearest neighbor analysis distances among individual outbound paths. Node support in the tree was assessed with the approximately unbiased test; clades with at least 95 % support are indicated by *rectangles*. *Node labels* show bird identity (B), track identity (T) and collection year (Y) and *are color*-*coded* by breeding stage (incubation in *red*, chick rearing in *blue*). The *vertical axis* shows the Euclidian distance between NNA distances

**Fig. 2:**
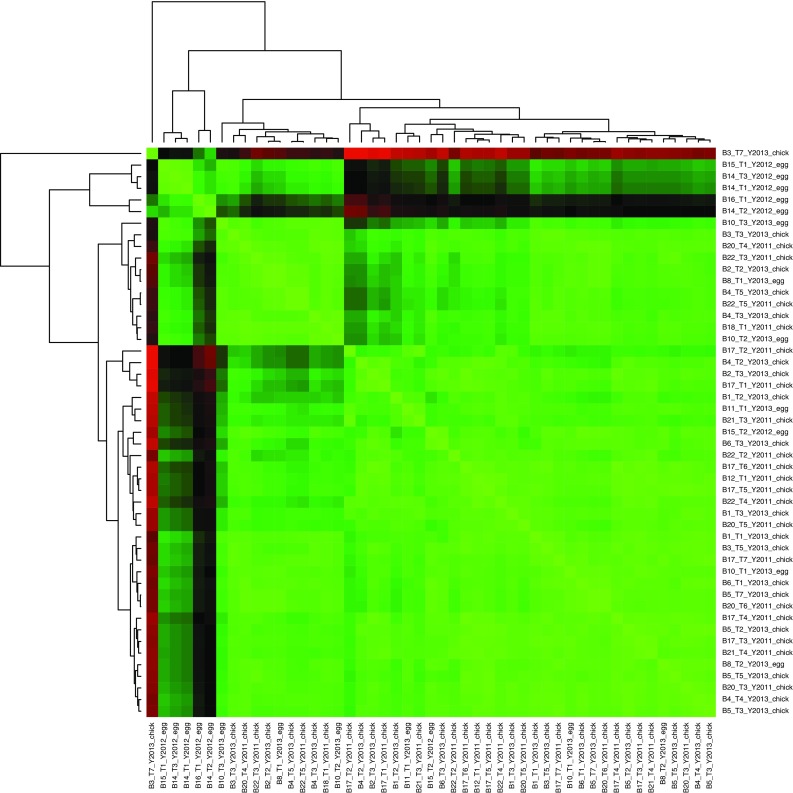
Heatmap of the nearest neighbor analysis (NNA). The NNA was performed on the sum of the longitude and latitude of the outbound trips, and the Euclidian distance was used to obtain a distance matrix of pairwise distances among the trips. This symmetric distance matrix is here represented as a heatmap, with *green* and *red* indicating small and large distances, respectively (*black* is intermediate). Each row/column of this matrix is labeled with an arbitrary bird ID “B” that uniquely identifies each bird, followed by a trip ID “T,” collection year “Y” and stage, either incubation (“egg”) or chick rearing (“chick”). The dendrograms to the top and left of the heatmap (both are identical) depict the hierarchical clustering as described in the main text (see also Fig. 1)

The NNA confirmed that while there is no evidence for intra-individual route fidelity, the difference between the two types of foraging range was largely explained by breeding stage identity (incubation vs. chick rearing; Figs. 1, 2). Indeed, after excluding tracks with <10 position fixes (“Methods”), 49 outbound tracks were analyzed. Of these, 97 % (36 out of 37) of chick-rearing tracks were grouped together (node A in Fig. 1: AU support = 86 %) with only one chick-rearing track being misclassified (Bird 3 Track 7). As a result, foraging tracks during chick rearing are highly similar (any AU support value >70 % is considered significant at the 5 % level; Suzuki and Shimodaira 2006). However, during incubation, only 42 % of tracks (5/12) were grouped together and more incubation tracks were misclassified than were chick-rearing tracks. Incubating birds therefore showed less track similarity than chick-rearing birds.

To test whether this pattern could be due to longer trips during incubation, we first examined average trip durations and lengths. The global mean (±SD) foraging trip duration was 16.6 ± 13.5 h (range 1.4–56.7 h) and went on average as far as 27.4 ± 8.6 km from the colony (range 17.1–54.8 km) per trip. Indeed, incubating birds had longer mean trip durations (INC: 23.7 ± 12.9 h and CHICK: 14.6 ± 13.4 h; estimate = 1.02 ± 0.27, |ΔAIC| = 4.6) and ventured further from the colony (INC: 34.3 ± 9.6 km and CHICK: 25.49 ± 7.3 km; estimate = 9.19 ± 2.95, |ΔAIC| = 10.48) than chick-rearing birds.

**Fig. 3:**
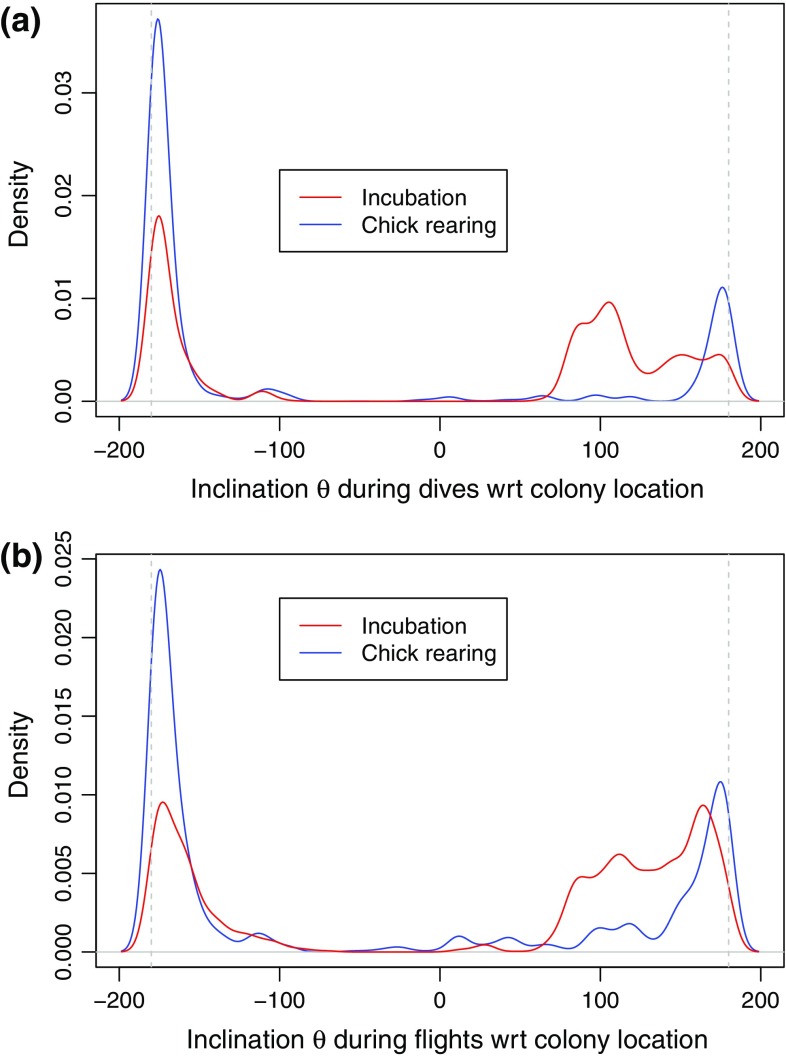
Distribution of bearing *θ* between the colony and GPS fixes. **a** For diving locations; **b** for fixes recorded during flight. Kernel density estimates during incubation are shown in red and during chick-rearing in *blue*. *Gray broken lines* indicate *θ* of 180°/−180°

**Fig. 4:**
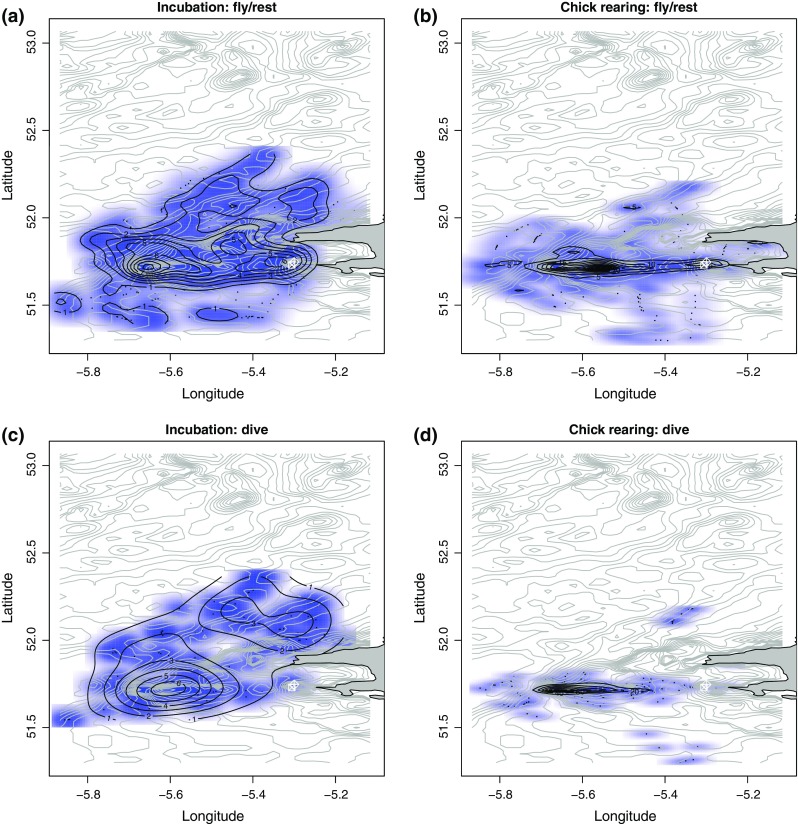
*Contour plots* of the kernel density distribution of diving and non-diving activity during the two breeding stages. Non-dive (flying/resting) densities are shown for **a** incubating and **b** chick-rearing birds, while corresponding diving densities are shown in **c** and **d**, respectively (*black lines*). Bathymetry lines are shown in *light gray*. The tip of Wales, UK, is shown in solid *dark gray*. Colony locations are shown by *crossed squares* and *diamond*

To confirm this pattern, we conducted an analysis of the distribution of bearings *θ* between the colony and where birds were either diving or flying. Figure 3 shows that while all birds dived and flew to or from the colony at an absolute angle |*θ*| of about 180° (east/west direction; see Fig. S2), incubating birds left the colony at angles varying between +90° and +160° (north/east north east; KS test; dives: *D* = 0.35, *P* < 2.2 × 10^−16^; flights: *D* = 0.28, *P* < 2.2 × 10^−16^). A density plot of the different activities shows that incubating birds travelled and dived over a larger and more dispersed area than chick-rearing birds (Fig. 4). This more focused area of chick-rearing birds’ activity was visually defined as our ROI, extending between approximately −5.73° and −5.3° of latitude and 51.65° and 51.82° of longitude (Fig. S4).

### Primary productivity as a potential driver of foraging strategy shift

Our results show that there was no difference in PP during incubation between foraging within and outside the ROI (*t* = −0.63, *df* = 1591, *P* = 0.52), suggesting that search patterns are not driven by PP during incubation. However, PP is very significantly higher within the ROI than outside during chick rearing (*t* = −10.48, *df* = 889, *P* < 2.2 × 10^−16^, two-tailed *t* test: Fig. S5). Further testing suggests that the foraging shift to the ROI between the two breeding stages may be due to an increase in PP in the ROI (*t* = −3.84, *df* = 910, *P* = 6.7 × 10^−5^). These last two results remain significant even after applying the conservative Bonferroni correction for multiple tests (Sokal and Rohlf 2012, p. 239). A bootstrapping analysis further confirms that PP within the ROI is significantly higher than in the surrounding area (Fig. S6).

### Horizontal search patterns are scale dependent but not stage dependent

**Fig. 5:**
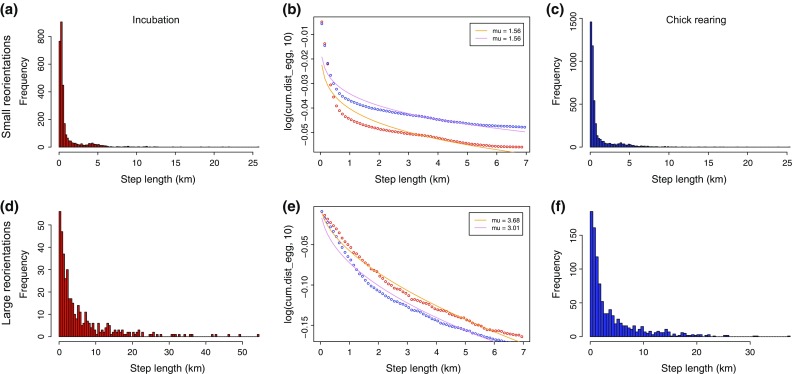
Reorientation patterns during flight during incubation (*red line*) and chick-rearing periods (*blue line*). **a** Histogram of small-scale reorientation step lengths in incubating birds, **b** distributions of small-scale reorientation step lengths during flight, **c** histogram of small-scale reorientation step lengths in chick-rearing birds, **d** histogram of large-scale reorientation step lengths in incubating birds, **e** distributions of large-scale reorientation step lengths during flight, **f** histogram of large-scale reorientation step lengths in chick-rearing birds. Estimates of $\mu$ are shown ± 1 SD

While the fit to the standard model (“Methods”) did not seem to be excellent (Fig. 5), its parameter estimates showed that reorientation is independent of the breeding stage but dependent on scale. Our estimates of scaling exponents suggest exponential motion at large angles (*μ* > 3 in both cases, all SDs <0.01, Fig. 5b) and heavy-tail movement at small angles (*μ* < 3 in both cases, all SDs <0.01, Fig. 5e).

## Discussion

Our data not only show that razorbills exhibit considerable variation in their foraging patterns during incubation, but also that they shift to a more focused pattern during chick rearing, targeting a ROI. Could these results be due to a device effect? Devices smaller than those used here (just 1–2 % of body mass) are known to impact foraging behavior in auks (Elliott et al. 2012; Vandenabeele et al. 2012; Wilson and Vandenabeele 2012), so that there is a possibility that the bio-loggers we used impacted where and how birds foraged. As in many studies, we were unable to have a true control to examine whether at-sea behavior had been affected or not by the attachment of loggers. Future studies would clearly benefit from quantifying device effects, which might be at the cost of reduced sample sizes due to a complex experimental design and limited physical resources at breeding colonies of wild animals. However, as we compared birds equipped with the same devices between two breeding stages, the observed behavioral shift is itself unlikely to be due to a device effect. To understand some of the potential drivers of the behavioral shift, we tested for an association with a shift in PP. We show that PP increases significantly in the ROI during chick rearing. Although it might be tempting to infer causation, it is important to bear in mind that PP is three to four trophic levels removed from razorbills’ diet, and these areas of high productivity are only ephemerally and locally available. In spite of this, our data are in line with previous studies suggesting that PP is the principal driver shaping foraging (Fauchald et al. 2000; Davoren et al. 2003). Other studies have documented changes in diet during chick rearing, changes that may be due to changes in food availability (Austin 1976; Custer and Pitelka 1978; Conner 1981; Jamieson et al. 1982; Robinson 1986; Annette 1987; Houston 1987; Petit et al. 1990; Sakai and Noon 1990). However, such diet changes could also (and non-exclusively) reflect diet changes between chicks and adults, as is often the case in other auks (Gaston et al. 1983; Davoren and Burger 1999; Wilson et al. 2004). It is also possible that adult diets differ between the two breeding stages, changing from lower (zooplankton based during incubation) to higher (fish-based during chick rearing) trophic levels, as shown to occur in other auks (Harris and Wanless 2011). While we did not determine stomach contents (to avoid additional disturbance to the animals), our results show that future studies should monitor diet in order to explain search strategies. Finally, it is possible that some seabirds exhibit intersex variations in their foraging behavior (e.g., Elliott et al. 2010). As we did not sex the studied birds (also to avoid additional disturbance to the animals), it is possible that some of the observed variations in foraging patterns may be due to sex differences between breeding stages.

Irrespective of their cause, behavioral shifts in aerial search patterns are known to occur at different life-history stages (Lopez–Lopez et al. 2013). Here, however, we found that razorbills’ search patterns were independent of the breeding stage. This suggests that behavioral flexibility is unlinked to search patterns in this species. Our results support the idea that individual behavioral flexibility in foraging is important and add new empirical evidence on how animals in general solve the problem of efficiently finding food resources in the wild (Gordon 1991).

The importance of behavioral flexibility is also consistent with our NNA results that showed that there is no intra-individual route similarity, so that there is no evidence supporting any connection between movement patterns and memory use. Consequently, our results suggest that foraging birds may read cues from their environment, including from conspecifics, to help improve their foraging efficiency. Indeed, because of the high correlation between the inbound and outbound flight directions during chick rearing (see Fig. 4a, b at |*θ*| ~ 180°), it is possible that birds departing from the colony on a foraging trip recognize returning birds that had a successful foraging trip and fly out toward this direction. Indeed, one demonstrated advantage of colonial breeding is that birds can detect a prey patch through either transmission of information (Weimerskirch et al. 2010) or a spatial concentration effect (Buckley 1997). This raises the question as to why only chick-rearing birds appeared to use recognition to decide on foraging flight bearings. Future studies of trajectory data recorded at higher temporal resolution may help further investigate the role of memory in the aerial search strategies of marine predators.

Our analyses reveal both scale-dependent and stage-independent foraging patterns (reorientations) in the context of stage-dependent foraging destinations (ROI during chick rearing). Such a stage-independent foraging pattern is unexpected considering the significant difference in foraging ranges between the two breeding stages. Indeed, although probabilistic search patterns are well documented (Edwards et al. 2007; Sims et al. 2008; Humphries et al. 2012), stage-independent foraging in a scale-dependent pattern has never been documented before. Two potential reasons may be that previous studies have focused mostly on a single breeding stage (Elliott et al. 2009) or that data from multiple stages were pooled (Humphries et al. 2012). Nevertheless, our results suggest that some animals such as breeding razorbills can have consistent search patterns across life-history stages while at the same time being able to adjust their foraging destinations flexibly in a seasonally changing environment.

## Electronic supplementary material

Below is the link to the electronic supplementary material.

Supplementary material 1 (PDF 3755 kb)
