# Supplementary material for: Foraging flexibility and search patterns are unlinked during breeding in a free-ranging seabird
Source: Mar Biol. 2016 Mar 14;163:72. doi: 10.1007/s00227-016-2826-x (PMC4791460; doi:10.1007/s00227-016-2826-x)
Supplement: Supplementary file 1 — Supplementary material 1 (PDF 3755 kb) [file 227_2016_2826_MOESM1_ESM.pdf]

Article title: Foraging flexibility and search patterns are unlinked during breeding in a free-ranging seabird

Journal name: Marine Biology

Akiko Shoji [1], Stphane Aris-Brosou [2], Ellie Owen [3], Mark Bolton [3], Dave Boyle [4], Annette Fayet [1], Ben Dean [1], Holly Kirk [1], Robin Freeman [5], Chris Perrins [4] and Tim Guilford [1]

[1]University of Oxford, Department of Zoology, Oxford, Oxfordshire, UK
[2]University of Ottawa, Department of Mathematics and Statistics, Ottawa, ON, K1N 6N5, Canada
[3]The Royal Society for the Protection of Birds, The Lodge, Sandy, Bedfordshire SG19 2DL, UK
[4]Edward Grey Institute of Field Ornithology, University of Oxford, Oxford, Oxfordshire, UK
[5]Institute of Zoology, Zoological Society of London, Regents Park, London, NW1 4RY, UK

Corresponding authors: Akiko Shoji (e-mail: akikosho@gmail.com).
Tim Guilford (email: tim.guilford@zoo.ox.ac.uk)

Supplementary text

Nearest Neighbor Analysis

To perform the Nearest Neighbor Analysis (NNA), individual foraging trips were first extracted. A foraging trip started when a bird was flying (*i.e.*, had a speed > threshold defined in Fig. S1) outside of a 2 km radius from the colony; the trip was completed when the bird flew back within the 2 km radius. Each trip was then subdivided between its outbound (the first part of the trip, out to sea) and inbound (return to colony) phases. The end of the outbound phase was defined by the point when the maximal distance from the colony was reached for each trip. The NNA presented in the main text focuses on the outbound phases to test route fidelity (i) within each bird, (ii) among birds and (iii) between breeding stages. This is because we were interested in foraging movement patterns, rather than homing patterns.

The spatial similarity between a focal trip t_f and a comparison trip t_c was computed as follows. Each outbound trip is a set of coordinates, longitudes and latitudes. At each positional fix, longitudes and latitudes were summed, for both the focal trip t_f and the comparison trip t_c. Trip similarity was then computed based on the match point (or *matching*) distance, which computes for each positional fix in the focal trip t_f its nearest neighbor in the comparison trip t_c. In the case of ties, only the neighbor with the smaller index (*i.e.*, that is closest to the colony) is given. The resulting vector of match distances is then averaged. Because this distance is asymmetric (the distance between trip t_1 and t_2 is not the same as that between trip t_2 and t_1), the mean of the two distances $d(t_f, t_c)$ and $d(t_c, t_f)$ is calculated. The resulting matrix of pairwise distances is therefore symmetric.

Taking inspiration from microarray studies, this matrix of pairwise distances can be visualized as a heatmap (Fig. S5) on which biclustering (clustering on both rows and columns) can be performed. To this end, a hierarchical clustering analysis (complete linkage method, based on Euclidean distances) was performed to quantify trip similarity. Trips with < 10 position fixes were excluded from this analysis. Significance was assessed by 1000 bootstrap replicates (BP; [1]) and by the Approximately Unbiased (AU; [2]) test using the pvclust package in R [3].

Two-dimensional density plots were based on the smoothScatter function in R that computes a binned 2D bivariate Gaussian kernel approximation based on Fast Fourier Transform convolutions.

Significance of the ROI

In order to assess the significance of the Region of Interest (ROI) in terms of primary productivity, a bootstrap procedure was implemented to select an area of the exact same size and perform the comparisons between: (i) ROI vs. not ROI during incubation, (ii) ROI vs. not ROI during chick rearing and (iii) Incubation vs. chick rearing within ROI, as in the main text.

Briefly, a position was selected at random from a bivariate uniform distribution to represent the lower left corner of the resampled ROI. The resampled ROI was then reconstructed by using the same dimensions as the original ROI. Primary productivity was then extracted for all positional fixes contained in this resampled ROI. This extraction was constrained on the breeding stage associated to each positional fixes. The vectors of primary productivities were then compared by means of a t-test. This entire procedure was repeated 10^5 times (for 10^5

random selections of resampled ROIs). In each case, the null hypothesis tested is about the absence of a difference between "treatments": if the original ROI is indeed truly special, then bootstrapped ROIs should show no significant differences.

References

[1] Efron B, Tibshirani R (1993) *An introduction to the bootstrap* (Chapman & Hall, New York) Vol. 57.

[2] Shimodaira H (2002) An approximately unbiased test of phylogenetic tree selection. *Systematic biology* 51:492–508.

[3] Suzuki R, Shimodaira H (2011) *pvclust: Hierarchical Clustering with P-Values via Multiscale Bootstrap Resampling* R package version 1.2-2.

Supplementary figures

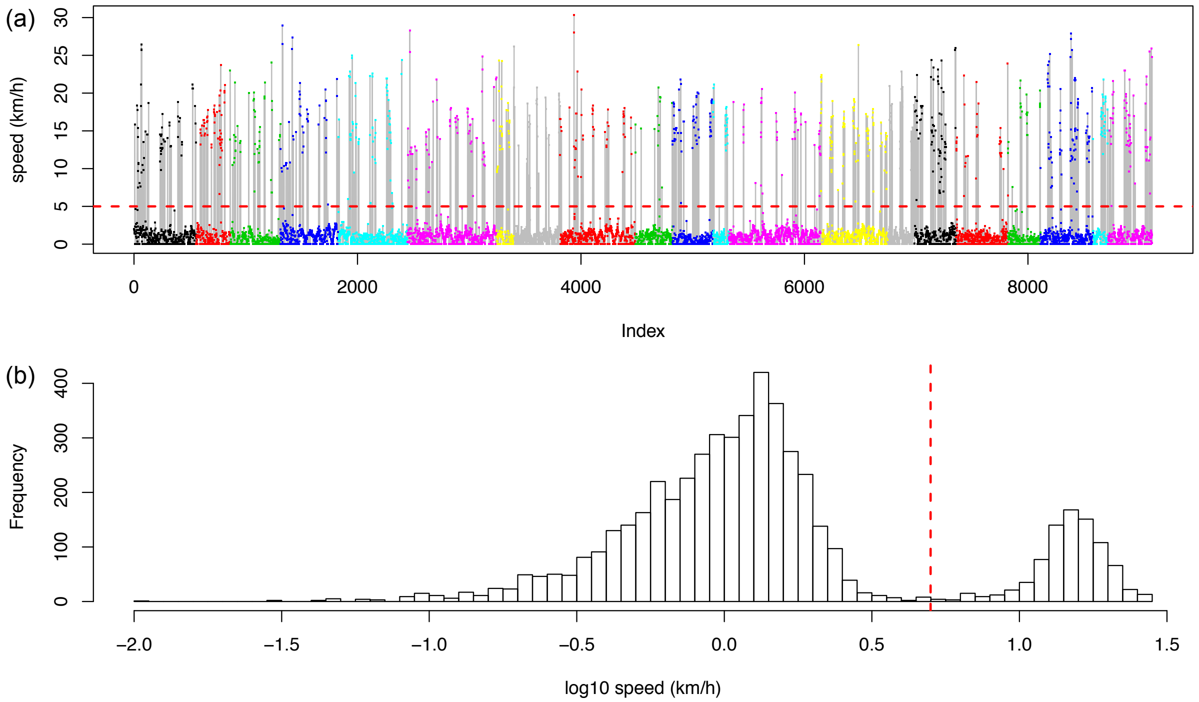

Figure S1: **Distribution of razorbills' speed for the 21 birds tracked.** (a) Speed is represented for each of the GPS logs. Alternating birds are represented in alternating colors (note that colors are recycled). Speed unit is in kilometer per hour (km/h). The red horizontal broken line is the 5 km/h threshold. (b) This speed threshold was determined according to the speed distribution, here represented on a $\log_{10}$ scale; the threshold is represented with a red vertical broken line, which separates the two modes of the speed distribution.

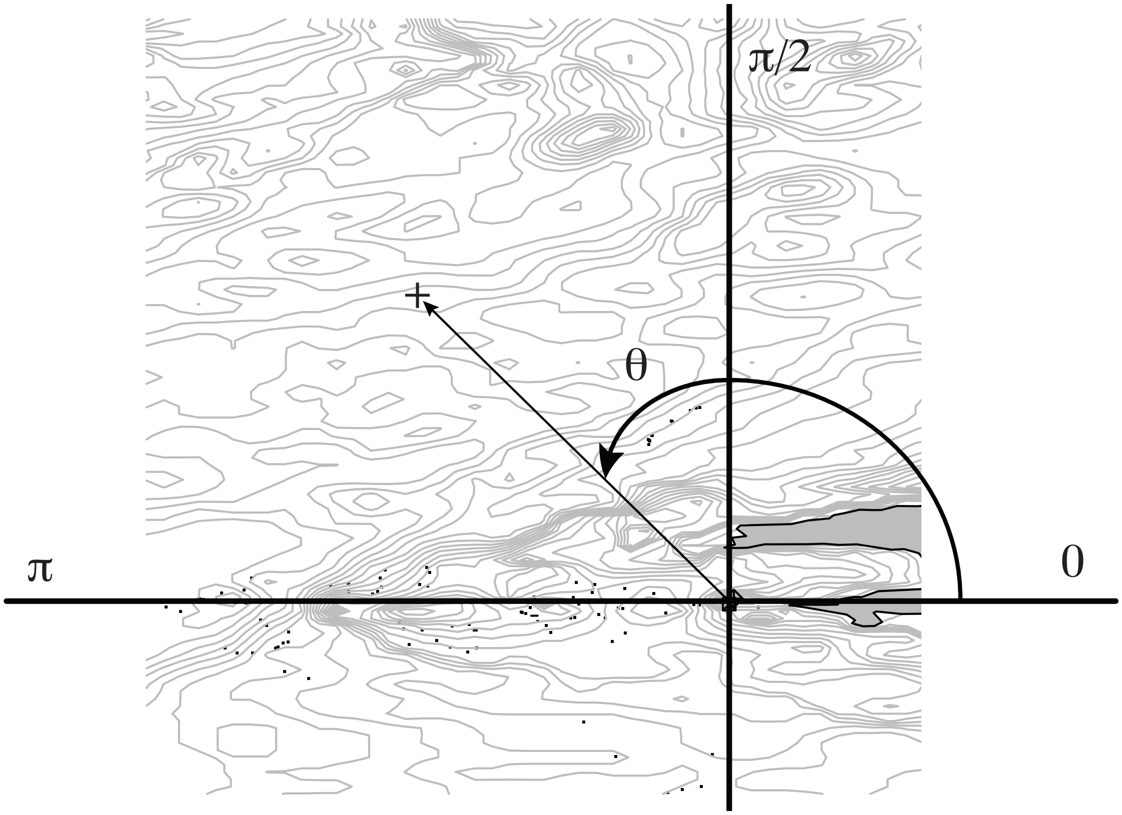

Figure S2: **Computation of bearings θ.** An orthonormal referential was centered on each colony location. The angle between the origin on the trigonometric circle and each position fix was calculated as described in the main text with the arctan2 function as $\theta = \arctan(lat, lon)$, where lat and lon are the difference in coordinates between the focal point and colony location for latitudes and longitudes, respectively. The arctan2 function is such that angles $\theta > \pi$ are converted into negative angles and therefore each bearing θ in Fig. 2 is such that $-\pi \leq \theta \leq \pi$.

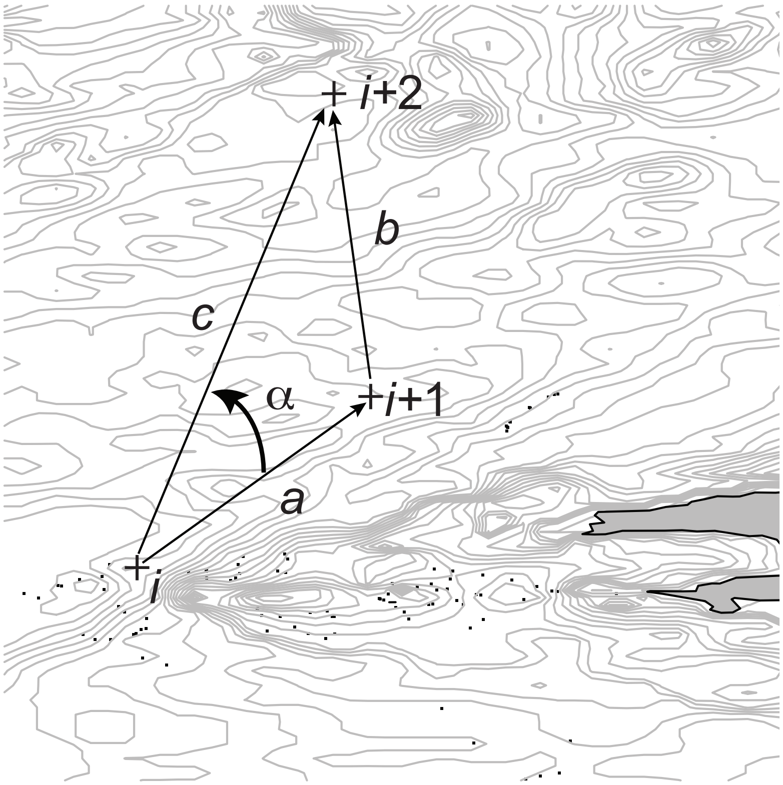

Figure S3: **Computation of reorientation angle α.** Three consecutive position fixes are required, here indexed as i, $i+1$ and $i+2$. Reorientation α is computed as described in the main text, based on segment lengths a, b and c: $\alpha = \arccos(\frac{b^2+c^2-a^2}{2ab})$. Note that α is always ≥ 0.

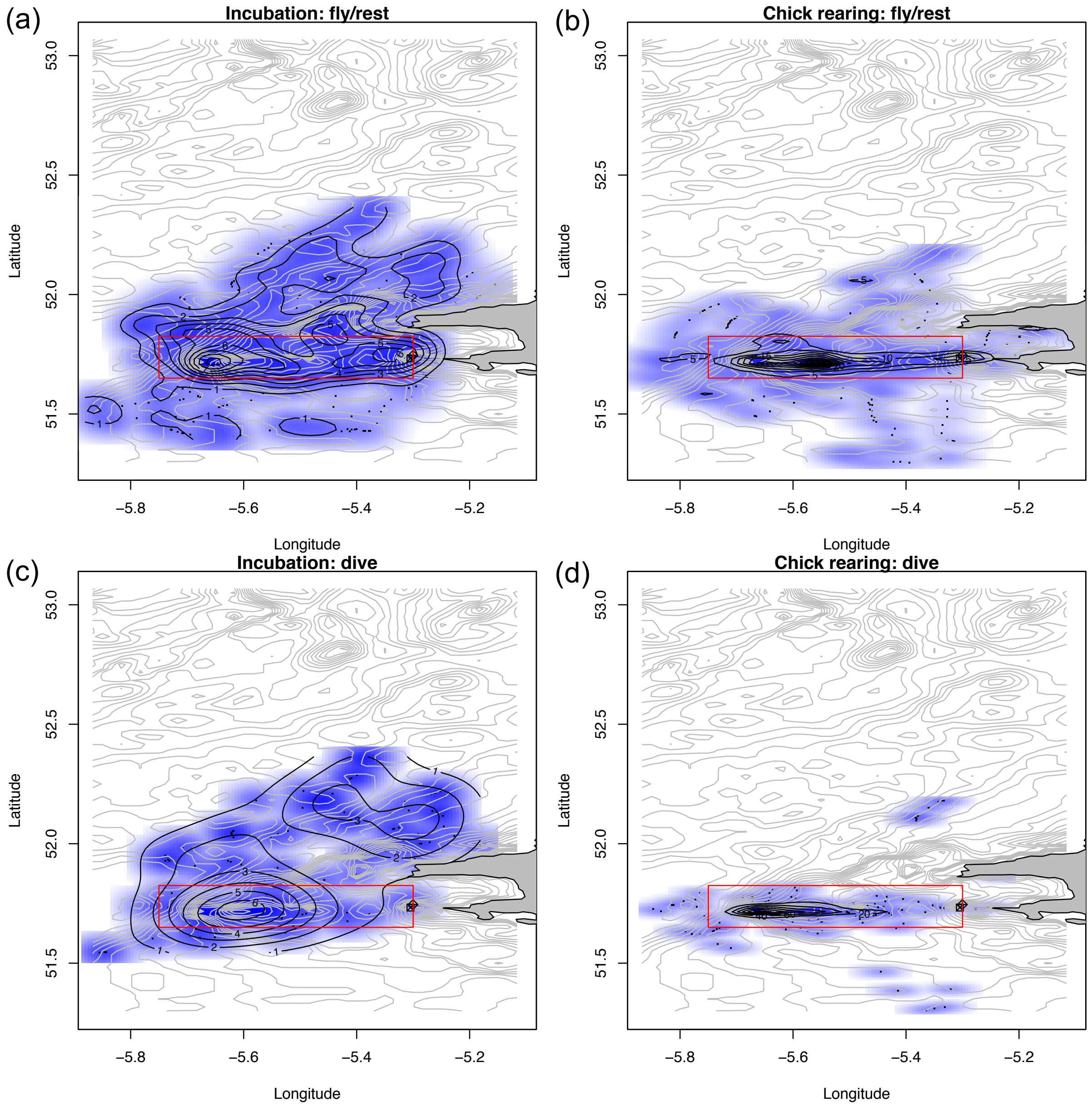

Figure S4: **Activity maps across the two breeding stages and flying/nonflying birds.** Nonflying activity maps are show in panels (a) and (b) for incubating and chick-rearing birds, respectively. Flying maps are show in panels (c) and (d) for incubating and chick-rearing birds, respectively. The shaded areas (in blue) show the density of each activity, where individual black dots show individual GPS (fly) or GPS/TDR (nonfly) logs. The Western tip of Wales is represented in dark gray, bathymetry is in light gray. Both colonies are shown. The position of the ROI is shown as a red rectangle.

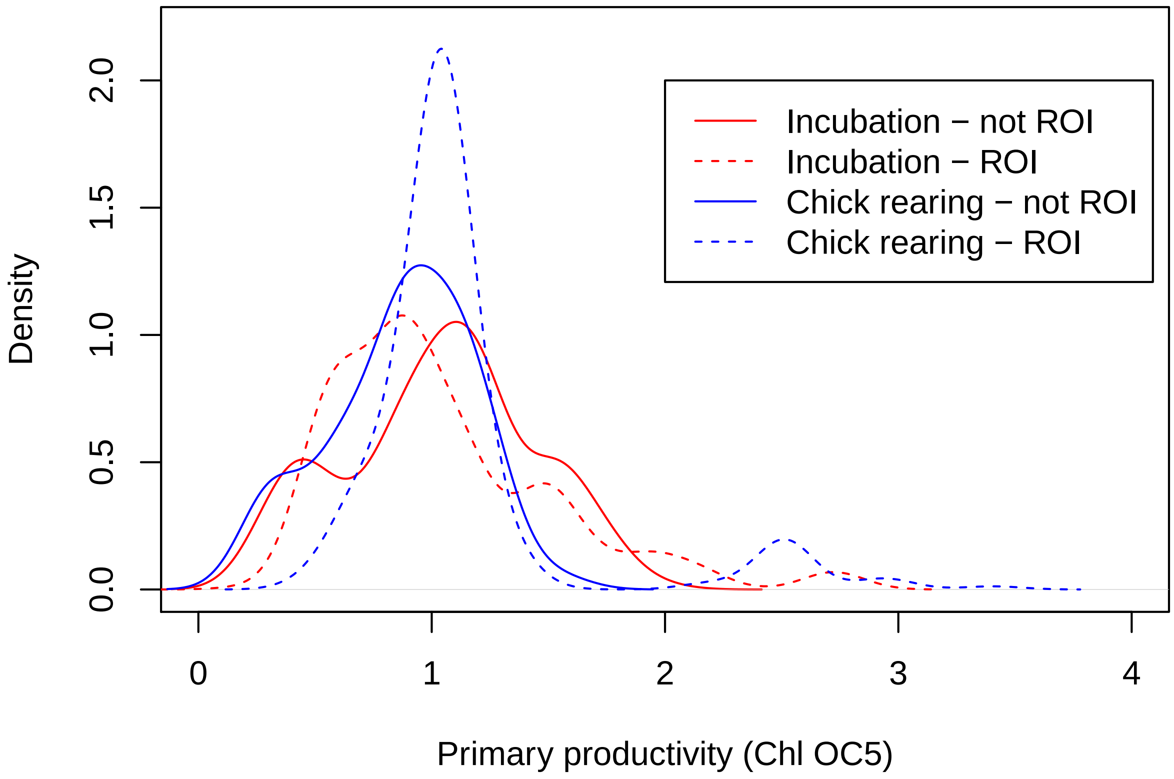

Figure S5: **Densities of primary productivity across the four conditions.** Densities of chlorophyll Chl OC5 are shown in red for incubating birds (solid lines: outside of ROI; broken lines: inside of ROI) and in blue for chick-rearing birds (solid lines: outside of ROI; broken lines: inside of ROI).

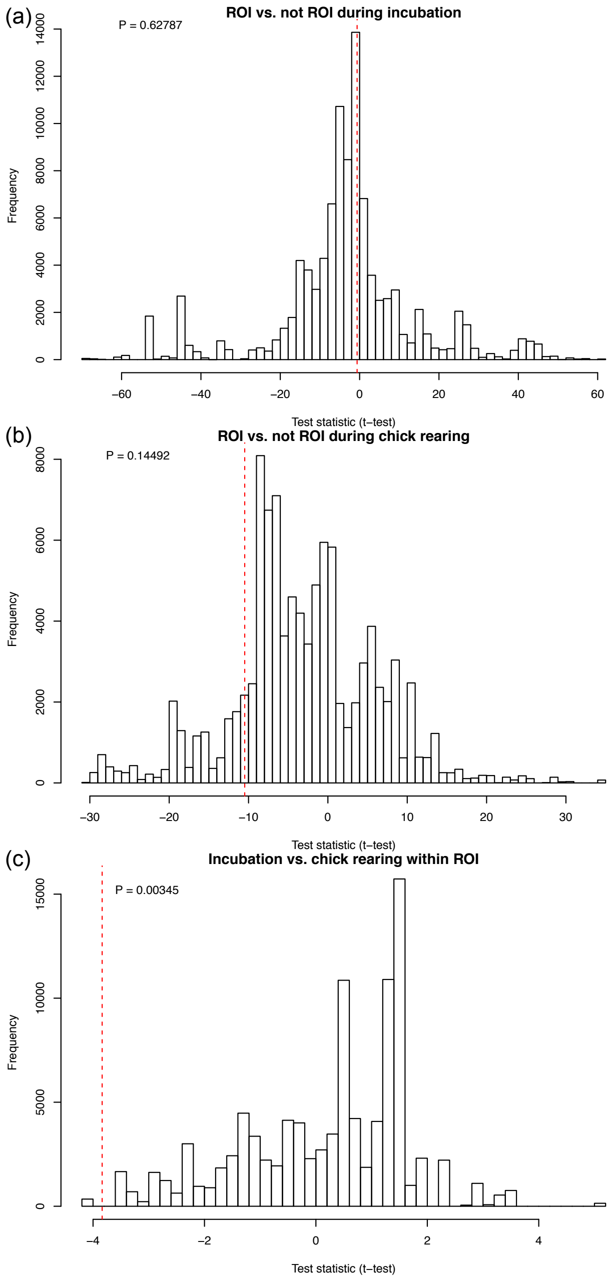

Figure S6: **Bootstrap analysis of significance of ROI for the three tests performed in the main text.** The three tests performed are for (a) ROI vs. not ROI during incubation, (b) ROI vs. not ROI during chick rearing and (c) Incubation vs. chick rearing within ROI. The observed values of the test statistic are shown in red (broken line) and the corresponding P-value is indicated (based on 10^5 replicates).
